# Review of the mite genus *Ololaelaps* (Acari, Laelapidae) and redescription of *O.formidabilis* Berlese

**DOI:** 10.3897/zookeys.853.29407

**Published:** 2019-06-06

**Authors:** Frédéric Beaulieu, Edwin Javier Quintero-Gutiérrez, Dorothee Sandmann, Bernhard Klarner, Rahayu Widyastuti, Orlando Cómbita-Heredia, Stefan Scheu

**Affiliations:** 1 Agriculture and Agri-Food Canada, Ottawa Research and Development Centre, Canadian National Collection of Insects, Arachnids and Nematodes, 960 Carling Ave., Ottawa, Ontario K1A 0C6, Canada; 2 Instituto Colombiano Agropecuario ICA, Subgerencia de Análisis y Diagnóstico, Grupo Red de Análisis y Diagnóstico Fitosanitario, Carrera 30 # 65-15, Manizales, Colombia; 3 University of Göttingen, J.F. Blumenbach Institute of Zoology and Anthropology, Untere Karspüle 2, 37073 Göttingen, Germany; 4 Institut Pertanian Bogor, Department of Soil Science and Land Resources, Damarga Campus, Bogor 16680, Indonesia; 5 Acarology Laboratory, Ohio State University, 1315 Kinnear Rd., Columbus, OH 43212, USA; 6 University of Göttingen, Centre of Biodiversity and Sustainable Land Use, Von-Siebold-Str. 8, 37075 Göttingen, Germany

**Keywords:** Gamasida, Indonesia, laelapid mites, rubber plantation, soil fauna, Sumatra

## Abstract

A species of laelapid mite, *Ololaelapsformidabilis*, is redescribed based on male and female adults from soil in Sumatra, Indonesia. This species is distinguished from other *Ololaelaps* species by its metapodal platelet narrowly fused with the parapodal plate and by its hologastric shield having two inverted-V-like ridges. The genus is redescribed based on a review of the literature and examination of specimens of some species. Valid species of *Ololaelaps* are listed and accompanied by notes on morphological characters to assist future revision of the genus.

## Introduction

Laelapidae is a large, ecologically diverse family of Mesostigmata, with several species described from Indonesia, including symbionts of bees (Krantz 1998, Delfinado-Baker and Baker 1999, Lundqvist 1999), tarantulas (Moraza et al. 2009), beetles (Berlese 1910), and ectoparasites of small mammals (Vitzthum 1926, Tenorio 1975, Hadi and Tenorio 1982). Yet, the dearth of information on soil-dwelling Laelapidae in Indonesia is striking.

The cosmopolitan genus *Ololaelaps* was initially proposed by Berlese (1904) for a cluster of species characterized by a well-sclerotized idiosoma and particularly a genitiventral shield fused with the anal shield. However, two of the five species originally included in the genus merely have a genital shield abutting a ventrianal shield, and were later transferred to genera that are now included in the family Ologamasidae (*Hydrogamaselluscoleoptratus* (Berlese), *Sessiluncusholostaspoides* (Canestrini), see Table 3). *Ololaelaps* species are found in soil and litter of wet meadows, forests and near water bodies (river banks, pond margins, seacoasts), as well as in rodent and insectivore nests, or less frequently on mammals themselves and in bird nests (Ryke 1962, Bregetova and Koroleva 1964). Laboratory rearing of a few species indicate that they are predators of nematodes, collembolans and other mites, and that they also scavenge on dead invertebrates (Hurlbutt 1958, Bregetova and Koroleva 1964, Walter et al. 1988). This genus includes 26 valid species names (Table 1) and only one has been recorded from Indonesia: *Ololaelapsformidabilis* Berlese, 1913. The genus clearly needs revision as the identity and taxonomic boundaries of most species are unclear, including most of the 11 species that have been described since the reviews of Ryke (1962) and Bregetova and Koroleva (1964).

The initial goal of this paper was to redescribe *O.formidabilis*, which was collected from soil in a rubber plantation (*Heveabrasiliensis* Müll. Arg.) near a lowland rainforest on the island of Sumatra, Indonesia. We took this opportunity to review the generic concept, based on the literature and examination of specimens of some species. We also present (1) a list of valid species of *Ololaelaps*, including notes on their most salient morphological features, and (2) a list of species that were previously classified as *Ololaelaps* but that have unclear taxonomic affinity (nomina dubia) or that now belong to other genera of Mesostigmata.

**Table 1.** List of valid *Ololaelaps* species (in bold) and synonyms (in regular font), type localities [and additional records], habitats and depository.

Species	Original genus	Type locality [other distribution records]	Type habitat [other records]	Type repository^1^	Notes and additional references (incl. for selected distribution records)
***bregetovae*** Shereef & Soliman, 1980: 81	*Ololaelaps*	EGYPT: Giza	debris	FAC	
***burdwanensis*** Bhattacharyya, 1978: 86	*Ololaelaps*	INDIA: Burdwan (West Bengal)	soil under grass beside pond	ZSI (presumably)	
***caucasicus*** Bregetova & Koroleva, 1964: 73	*Ololaelaps*	RUSSIA: near Kizlyar (Dagestan); ARMENIA: Yerevan^2^	litter of *Elaeagnus* (Russia), litter under ash tree (Armenia)	ZIN	Bregetova 1977a
*confinis* Berlese, 1904: 261 [?**syn. of *placentula***]	*Ololaelaps*	NORWAY	?	ISZA ^3^	Not illustr. by Berlese (1904); synonymy by Ryke (1962), accepted by Evans and Till (1966); it’s unclear (1) if types have been re-examined and therefore (2) if this syn. is valid (Bregetova and Koroleva 1964)
***dililoensis*** Marais & Loots, 1972: 31	*Ololaelaps*	REPUBLIC OF THE CONGO: Eala	soil	KMMA	
***expansus*** Ma, 2015: 95	*Pristolaelaps*	TAIWAN: Tainan	soil	AMMS	
*flavus* Ewing, 1909: 66 [**syn. of *placidus***]	*Laelaps*	USA: Arcola (Illinois)	under log	USNM (lectotype)	Lectotype designated by Hennessey and Farrier (1988)
***formidabilis*** Berlese, 1913: 82	*Ololaelaps*	INDONESIA: Semarang (Java); [Sumatra (this paper)]	? [forest litter]	ISZA ^3^	
*gamagarensis* Jordaan & Loots, 1987: 49 [**syn. of *mooiensis***]	*Ololaelaps*	SOUTH AFRICA: Gamagara River, Sishen	soil under grasses and reeds, on river bank	NWU	Synonymy by Nemati et al. (2018), based on types comparison and intraspecific variation of specimens from Iran
*haemisphaericus* Koch, 1839b: 16 [?**syn. of *sellnicki***]	*Iphis*	GERMANY	marshy meadows	?	Recognized as *Ololaelaps* by Sellnick (1940: 69) and as *Stylochirus* (Ologamasidae) by others (see Table 3 and main text for details)
*halaskovae* Bregetova & Koroleva, 1964: 81 [**syn. of *venetus***]	*Ololaelaps*	RUSSIA (widespread locations); UKRAINE (Zakarpattia Oblast); MOLDAVIA: Egorovka^2^	litter in meadows and forests; on small rodents or in their nests	ZIN	Synonymy by Evans and Till (1966)
***hemisphaera*** Berlese, 1916b: 303	*Ololaelaps*	USA: Columbia (Missouri^4^)	litter	ISZA ^3^	Farrier and Hennessey 1993
***holaspis*** Oudemans, 1902b: 53	*Hypoaspis*	ITALY: Sanremo	litter	RMNH	
***interruptus*** Karg, 1994: 186	*Pseudoparasitus*	ECUADOR (Galápagos Islands): Cerro Banderas, 4 km NE of Santa Rosa, Santa Cruz island	litter of *Miconia* sp., in a cave	ZMB	
***leptochelae*** Karg, 1994: 187	*Pseudoparasitus*	ECUADOR (Galápagos Islands): near El Puntudo, Santa Cruz island	moist litter in fern-sedge zone	ZMB	
*magnichelas* Ewing, 1909: 65 [**syn. of *placidus***]	*Laelaps*	USA: Muncie (Illinois)	moss	USNM (lectotype)	Lectotype designated by Hennessey and Farrier (1988)
***mooiensis*** Ryke, 1962: 126	*Ololaelaps*	SOUTH AFRICA: Mooi River, Potchefstroom; [ANGOLA, IRAN]	damp soil on river bank; [soil, litter]	NWU	Marais and Loots 1972, Halliday 2005, Nemati et al. 2018
***nasri*** Hassan, 1989: 593	*Ololaelaps*	EGYPT: Kafr Shokr	debris under citrus trees	?	
***obovatus*** Womersley, 1960: 33	*Pristolaelaps*	AUSTRALIA: Koroit (Victoria)	?	SAM	
***paratasmanicus*** Ryke, 1962: 127	*Ololaelaps*	NEW ZEALAND: Dunedin; [CHINA: Kunming]	bracken	NHMUK	Ma 2015
***placentula*** Berlese, 1887: 3	*Laelaps*	ITALY: Vallombrosa; [widespread in Europe; CHINA, RUSSIA, USA, CANADA]	moss; [litter in forests and meadows, nest of small mammals]	ISZA ^3^	Bregetova and Koroleva 1964, Lapina 1976, Farrier and Hennessey 1993, Luxton 1998, Gwiazdowicz and Klemt 2004, Salmane and Kontschán 2005, Bai and Ma 2014
***placidus*** Banks, 1895: 128	*Laelaps*	USA: near Roslyn (New York); [CANADA]	wet moss; [litter]	MCZ	Lectotype designated by Hennessey and Farrier (1988); Farrier and Hennessey 1993
***platensis*** Berlese, 1916a: 166	*Ololaelaps*	ARGENTINA: La Plata	?	ISZA ^3^	
***rectagoni*** Karg, 1993b: 269	Pseudoparasitus (Ololaelaps)	ECUADOR (Galápagos Islands): south of Wreck Bay, San Cristóbal island	moist and salty litter	ZMB	
***sellnicki*** Bregetova & Koroleva, 1964: 77	*Ololaelaps*	RUSSIA, UKRAINE, LITHUANIA^2^; [widespread in western parts of Eurasia]	wet meadows, stream banks, coastal habitats, alpine meadows, rodent nests	ZIN	Bregetova and Koroleva (1964) proposed the name *O.sellnicki* to represent *O.haemisphaericus* (Koch 1839b) (see main text); Evans and Till 1966, Solomon 1968, Beron 1974, Lapina 1976, Kavianpour et al. 2017
***sinensis*** Berlese, 1923: 252	*Ololaelaps*	CHINA: near Beijing	?	ISZA ^3^	Originally described as O.venetusvar.sinensis; Ryke (1962)Bai et al. (1996) and Karg (1978) use *sinensis* at species level; types may never have been re-examined
***sitalaensis*** Bhattacharyya, 1978: 84	*Ololaelaps*	INDIA: Sonarpur (West Bengal)	litter under *Pistiastratiotes* at pond margin	ZSI (presumably)	
***tasmanicus*** Womersely, 1956: 571	*Pristolaelaps*	AUSTRALIA: Tasmania; [USA: Hawaii; NEW ZEALAND]	strawberry plants; [moss, soil, on a rat]	SAM	Womersley 1960, Tenorio 1982
***translineatus*** Barilo, 1991: 15	Pseudoparasitus (Ololaelaps)	UZBEKISTAN: Baysun	turf of [urban] park	SIZK	
***ussuriensis*** Bregetova & Koroleva, 1964: 75	*Ololaelaps*	RUSSIA (Primorsky Territory)^2^; [CHINA]	on small rodents, in their nests, or soil	ZIN	Bregetova 1977a, Ren and Guo 2008
***venetus*** Berlese, 1903: 14 [?jun. syn. of *placidus*]	Laelaps (Hypoaspis)	ITALY: Veneto^3^; [widespread in Europe and parts of Asia]	moss; [see records for *O.halaskovae*]	ISZA ^3^	Laelaps (H.) venetus was proposed by Berlese (1903) for specimens misidentified as *Laelapstumidulus* (Koch) in Berlese (1889: 5); as syn. of *O.placidus* in Hennessey and Farrier (1988); Luxton 1998, Gwiazdowicz and Klemt 2004, Ren and Guo 2008
***wangi*** Bai, Gu & Wang, 1996: 74	*Ololaelaps*	CHINA: Southern Yinchuan; [SOUTH KOREA]	decaying *Zeamays*; [grassland soil]	EDC	Keum et al. 2017

? indicates unknown or uncertain data. **^1^**Type repository: AMMS – Academy of Military Medical Sciences, Institute of Microbiology and Epidemiology, Beijing, China; EDC – Institute of Endemic Disease Control, Ningxia Hui Autonomous Region, China; FAC – Faculty of Agriculture, Cairo University, Giza, Egypt; ISZA – Istituto Sperimentale per la Zoologia Agraria, Firenze, Italy; KMMA – Koninklijk Museum voor Midden-Afrika, Tervuren, Belgium; MCZ – Museum of Comparative Zoology, Harvard University, Cambridge, USA; NHMUK – The Natural History Museum, London, United Kingdom; NWU – North-West University, Potchefstroom, South Africa; RMNH – Naturalis Biodiversity Center, Leiden, The Netherlands; SAM – South Australian Museum, South Australia, Australia; SIZK – Schmalhausen Institute of Zoology of National Academy of Sciences of Ukraine, Kiev, Ukraine; USNM – United States National Museum, Beltsville, USA; ZIN – Zoological Institute of the Russian Academy of Sciences, St. Petersburg, Russia; ZMB – Museum für Naturkunde, Berlin, Germany; ZSI – Zoological Survey of India, Kolkata, India. **^2^**Localities based on type series. **^3^**Also based on Castagnoli and Pegazzano (1985), which provide information on specimens kept at the ISZA (= “Berlese Acaroteca”). **^4^**The type locality “Columbia (N.A.)” indicated in Berlese (1916b) is probably Columbia, Missouri (USA) because at least three species described in Berlese (1916) are from “Columbia (N.A.)” and later taxonomic revisions published by various authors indicate that the type locality for those species is Columbia, Missouri.

## Materials and methods

This study is part of a larger investigation on arthropods of Indonesia within the framework of the interdisciplinary project “Ecological and socioeconomic functions of tropical lowland rainforest transformation systems (Sumatra, Indonesia)” – EFForTS. For details on the study region and the experimental design, see Drescher et al. (2016).

Soil and litter samples were taken, using a spade, from rubber plantation plots at the rainforests of Bukit Duabelas (National Park) and Harapan (National Forest), Jambi Province, Sumatra (see “Material examined” section for details on localities). Samples represented 16 × 16 cm of litter and soil taken down to a 5-cm depth. Mites were extracted from samples using a modified high-gradient canister method (Kempson et al. 1963). Mites were stored in 70% ethanol until clearing in 55% lactic acid and slide-mounting in Hoyer’s medium. Specimens were dissected prior to slide-mounting to separate the gnathosoma from the idiosoma.

Photographs and measurements were made using a compound microscope (Nikon Eclipse Ci or Leica DM5500B) equipped with phase contrast or differential interference contrast and connected to a computer-controlled digital camera (Sight Ds-L3 or Leica DMC4500). Most images were captured in stacks (with focal depth manually or electronically controlled). Selected images were combined using Zerene Stacker version 1.04 or Helicon Focus 6.7.1 Pro (Helicon Soft Ltd., 2000). Digital drawings were prepared using Adobe Illustrator, version CC 2015 (19.0.0), based on mite photographs that were first imported into the software.

All measurements are given in micrometers (μm) and presented as ranges (minimum–maximum). Lengths of shields were measured along their midlines, and widths at the widest point except for the sternal shield, measured at level of setae st2. Legs were measured from proximal margin of the coxa to the tip of tarsus, excluding ambulacrum (stalk, claws, pulvillus), and corniculi from their apex to the midpoint of their internal base. Spermatodactyl was measured from its point of departure from the movable digit to its apex. Notations of structures and idiosomal chaetotaxy generally follow Lindquist and Evans (1965), as slightly modified by Lindquist (1994); leg chaetotaxy follows Evans (1963) and Evans and Till (1965). Notations of idiosomal pore-like structures, as gland openings and poroids (proprioceptors, often called ‘lyrifissures’), follow mostly that of Athias-Henriot (1971, 1975) and secondarily Johnston and Moraza (1991), as applied by Kazemi et al. (2014) to Laelapidae.

Specimens of *O.formidabilis* are deposited in LIPI (Indonesian Institute of Science), Cibinong, Indonesia; the SMNG(Senckenberg Museum), Görlitz, Germany; and the CNC(Canadian National Collection of Insects, Arachnids and Nematodes), Ottawa, Canada.

Additional photos of the species are digitally deposited in the online database available at ecotaxonomy.org.

The diagnosis and description of the genus were prepared after consultation of previous diagnoses of the genus (Womersley 1956, Ryke 1962, Bregetova and Koroleva 1964, Evans and Till 1966, Bregetova 1977a, Keum et al. 2017, Joharchi et al. 2018) and species descriptions, as well as examination of specimens at hand of three described species (*O.formidabilis*, *O.placidus*, *O.placentula*), two tentatively identified species from Colombia (O.nrdililoensis) and Iraq (O.nrmooiensis) and at least three undescribed species from North America and Costa Rica. The species list in Moreira (2014) was consulted to aid in tracking species descriptions. Species authorships are presented in Table 1.

### Taxononomic accounts

#### Genus Ololaelaps

Taxon classificationAnimaliaMesostigmataLaelapidae

Berlese, 1904: 260

Pristolaelaps Womersley, 1956: 571. Synonymy by Ryke (1962).

##### Type species.

Laelaps (Hypoaspis) venetus Berlese, 1903

##### Diagnosis

(adult male and female, unless stated).Well-sclerotized hypoaspidine laelapid with a hologastric (genitiventrianal) shield in female, bearing 3–5 pairs of preanal setae (plus st5), as well as the following character states: dorsal shield covering entirely idiosoma dorsally, narrowly to broadly extending onto venter; bearing 39 or slightly fewer pairs of slender setae, including px2–3 and often one Jx. A pair of well-sclerotized presternal platelets. Female with seta st4 on sternal shield or on soft cuticle (or putatively on endopodal plate). Peritrematal shield free posteriorly or variously (narrowly) fused with hologastric and/or parapodal shields, via metapodal platelet; metapodal platelet free or variously fused to above-mentioned shields; parapodal plate well-developed, subtriangular. Soft opisthogastric cuticle with 5–10 pairs of setae. Male holoventral shield broad, fused to parapodal-exopodal plates, sometimes also to peritrematal shield. Gnathotectum convex, with few to numerous fine denticles; deutosternal groove with six rows of 1–10 denticles; female cheliceral movable digit with two teeth (rarely more), fixed digit with 3–5 (exceptionally 8); palp-apotele three-tined, third tine reduced. Leg chaetotaxy normal for Laelapidae; setae generally slender.

##### Description.

***Dorsal idiosoma.***Dorsal shield relatively large (435–800 in female), broadly oval to narrowly suboval (length/width ratio 1.2–1.8), completely covering idiosoma dorsally, barely to moderately extending ventrally (this can be determined most accurately before slide-mounting); shield smooth (except for fine granulation or punctuation) to strongly reticulate; shield’s ventral extension (‘epipleura’ of Bregetova and Koroleva (1964)) smooth to reticulate (sometimes in contrast to smooth dorsal region of shield); shield with a delineated marginal strip along its edge. Dorsal shield bearing 39 pairs of simple, slender, almost hair-like setae, short to moderately long, including px2–3, and often one unpaired median seta (Jx) inserted at a level between J2 and J3 (Table 2); sometimes fewer than 39 pairs of setae, with z1 (absent in *O.sellnicki*), z3 (see Evans and Till 1966), or setae in r or S series apparently absent; shield never hypertrichous; setae slender and smooth, occasionally with a few light barbs on Z5 and J5 (Jordaan and Loots 1987). Shield with 16 pairs of poroids and four or five pairs of gland openings (based on Bregetova and Koroleva (1964) and specimens examined, representing a few species only): gd1, gd2 (sometimes absent), gd4 (usually conspicuous, on or near shield margin), gd6, gd9.

***Ventral idiosoma.***Tritosternum normal, with two pilose laciniae. Presternal region with a pair of sclerotized platelets, wedge-shaped to subrectangular, lineate (typically with 2–4 transversal lineae); typically an additional, poorly sclerotized area, lineate and granulate, anteriorly or anteromesally adjoining each platelet. Female sternal shield as long as or longer than wide, sometimes wider than long; shield length/width ratio 0.6–1.8; Shield posterior margin straight, slightly to moderately concave, or sometimes convex; shield reticulate, smooth in its posterior fourth or fifth, bearing 3–4 pairs of simple setae and 2–3 pairs of poroids, therefore sometimes including seta st4 and poroid iv3; seta st4 on sternal shield (near or on its posterolateral edge), on soft cuticle, or apparently on endopodal plate (Table 2; see Discussion). Female hologastric shield broad, laterally extending to or beyond margin of parapodal (or adcoxal, Bregetova (1977a)) plate, overlapping sternal and endopodal shields, bearing usually five pairs of preanal setae (JV1–3, ZV1–2) in addition to seta st5, occasionally only three or four pairs of preanals (JV3 and/or ZV2 off shield) or exceptionally six (ZV3 apparently on shield in *O.rectagoni*); shield setae usually moderately long, sometimes short; hologastric shield ornamented with reticulation, cells polygonal, scale-like or elongate transversally (note that shield reticulation is not drawn for some species in Ryke (1962), but probably present; compare *O.mooiensis* in Ryke (1962) vs Marais and Loots (1972)); circumanal setae shorter than preanals, and postanal usually shorter than paranal setae; cribrum typically with 2–3 rows of spicules. Endopodal plate besides coxae III–IV well-developed (appears reduced in *O.dililoensis*, but the portion of endopodal plate that is overlapped by hologastric shield may have been overlooked); plate free, more or less contiguous with sternal shield (or apparently fused to it, e.g., *O.expansus* (Ma 2015)) or slightly overlapped by it. Peritrematal shield well-developed, fused to dorsal shield anteriorly, usually free posteriorly, reaching approximately posterior margin of coxa IV, occasionally only mid-coxa IV, or moderately surpassing coxa; sometimes narrowly connected to hologastric shield and/or parapodal element; peritrematal shield posteriorly bifid in some species (*O.interruptus*, *O.leptochelae*, and an undescribed species from North America). Peritreme narrow, usually reaching anteriorly level of coxa I, sometimes slightly less. Parapodal plate well-developed, subtriangular, with outer margin convex (especially when peritrematal shield free and not extending beyond coxa IV) or straight (typically when peritrematal shield extended posteriorly or fused to hologastric shield); parapodal exceptionally not produced in *O.rectagoni* (Table 2) and an undescribed species from Costa Rica; parapodal posteriorly free, more or less abutting hologastric shield, or narrowly fused to hologastric and/or peritrematal shields, via metapodal element as connecting ‘bridge’. Metapodal platelet entirely free, suboval to strip-like, or variously fused to hologastric shield, parapodal and/or peritrematal plates. Exopodal strip well-developed, fused to parapodal element posteriorly, and anteriorly to sternal shield between coxae I–II. Soft opisthogastric cuticle surrounding shield with 5–10 pairs of simple setae, often including 1–2 pairs of r-R setae isolated at level near parapodal plate; never hypertrichous. Male holoventral shield fused to parapodal-exopodal elements, sometimes also to peritrematal shield, bearing 3–5 preanals (JV1–3, ZV1–2; JV3 and ZV2 sometimes off shield, e.g., *O.ussuriensis*); metapodal element merged with holoventral shield.

***Gnathosoma.***Gnathotectum with subtriangular to rounded margin, usually finely denticulate, may appear smooth when denticles sparse or (possibly) absent. Deutosternal groove of moderate, regular width, or slightly tapering posteriorly, with six (occasionally seven, and rarely five) rows of denticles, each row bearing 1–10 denticles, most of the rows with 3–7 denticles; denticulate rows usually preceded by a smooth ridge anteriorly, and sometimes also posteriorly. Corniculi horn-like, of moderate length. Internal malae with two pairs of long projections, median pair fimbriate on its basal portion, lateral pair smooth or branched or fimbriate in its apical portion; lateral projection absent in males (and apparently in the female of *O.sitalaensis*). Palptarsal claw three-tined, third tine reduced. Chelicerae of moderate length, chelate-dentate; female movable digit with two moderately-sized teeth, rarely more (two additional small teeth between the two typical large teeth in *O.interruptus*; Table 2); fixed digit with 3–5 teeth, variously sized, rarely more (eight in *O.leptochelae*), including a subapical, laterally offset tooth (gabelzhan); male digits each with a single tooth; pilus dentilis setiform; arthrodial process a simple corona. Male spermatodactyl 0.7–2.0 × as long as movable digit, from its departure from edge of digit; more or less straight or variously bent; junction between spermatodactyl and movable digit straight to strongly angled (*O.translineatus*); duct inside spermatodactyl straight or sinuous. Chaetotaxy of subcapitulum and palps normal for Laelapidae (sensu Evans and Till 1965).

***Legs.***Chaetotaxy normal for Laelapidae (sensu Evans and Till 1965); most setae slender; ventral and/or subapical setae of tarsi II–IV usually moderately thickened, sometimes lateral setae too (e.g., al2, pl2–3 of tarsus IV); setae on other leg segments occasionally thickened (e.g., pd2, ad3 on femur I, pd on femur III in *O.placentula*; also dorsally on femur IV in *O.mooiensis* (Jordaan and Loots 1987)). Males of some species with a spine-like seta on femur II (*O.translineatus*); pv thickened on genu or tibia III (in undescribed species); a ventral spine on tarsus II, apparently representing pv2 (position shifted proximad) (e.g., *O.venetus*, *O.placentula*, *O.ussuriensis*); or with cuticular tubercles on various leg segments (femur and genu of *O.placentula*, *O.ussuriensis*). Ambulacra I–IV with well-developed paired claws and pulvillus.

***Spermatheca.***

Spermathecal ducts well-sclerotized and discernable in some species.

**Table 2.** Some diagnostic features of valid *Ololaelaps* species based on the literature, except for a few species for which type (**) or voucher (*) specimens were examined. Species are sorted in groups based on shared features, mainly the various fusion of shields ventrally (groups may or may not reflect relatedness).

Species	Shared features (mostly fusion of shields^1^)	Dorsal shield ornamentation^1^	Epipleura^2^ ornament.	Dorsal seta *Jx*	Insertion of *st4*^3^	Other features^1^	Notes and references (redescriptions)
***venetus***	(1) all shields (HOLOG + METAP + PERIT + PARAP) narrowly fused together; (2) spermatod. with sinuous duct; (3) spermathecae well-sclerotized, distinctive	smooth with sculptured areas anteriorly (Evans and Till’s text)	smooth	1	stern.	*JV3*, *ZV2* setae sometimes off HOLOG	Ryke 1962, Bregetova and Koroleva 1964, Evans and Till 1966, Bregetova 1977a
placidus*		smooth except light reticul. near ant. margin	smooth	0–1	stern.	as above	Hennessey and Farrier 1988, F.B. pers. obs.
***sellnicki***		as *venetus*; reticul. visible only when freshly moulted (Bregetova and Koroleva 1964)	smooth?^4^	1	stern.	*JV3*, *ZV2* off HOLOG; *z1*, *z3* absent	Evans and Till 1966, Solomon 1968, Bregetova 1977a, Kavianpour et al. 2017; also Sellnick (1940), as *O.haemisphaericus*
***hemisphaera***	HOLOG + METAP + PERIT fused [PARAP apparently free]	?	lineate-reticulate?	?	soft cut.?	broad idiosoma	not illustr. in Berlese (1916b); partly illustr. in Ryke (1962), possibly based on Berlese’s types or drawings (see Ryke’s introduction)
***interruptus***	(1) HOLOG + METAP + PERIT narrowly fused [PARAP clearly free]; (2) PERIT notched post.	?	?	1	soft cut.	MD with 2 small teeth in-between the 2 standard teeth; broad idiosoma	
***leptochelae***		?	?	?	?	FD with a total of 8 teeth	
***burdwanensis***	HOLOG + METAP + PARA narrowly fused [PERIT free]	?	lineate-reticulate	1	soft cut.?		
***translineatus***		smooth?	lineate-reticulate	1	soft cut.?	sternal shield with transverse ridge; spermatod. at 90° angle from MD; spermath. distinctive	similar to *O.burdwanensis*
***wangi***		smooth except lineate anteriorly	lineate-reticulate	0	soft cut.?	only 2–4 deutosternal denticles / row	similar to *O.burdwanensis*; Keum et al. 2017
formidabilis*,**	only METAP + PARAP fused	light reticul.; lighter and sparser anteriorly	reticulate	0–1	soft cuticle	HOLOG with inverse V-shaped ridges; spermatod. elongate; spermath. not discerned	*O.formidabilis* sensu Ryke (1962) differs: METAP partly fused to HOLOG, not to PARAP
***caucasicus***	only HOLOG + METAP (partly to completely) fused	similar to *placentula* or *ussuriensis*?	lineate-reticulate	0–1	stern. or soft cut.	broad idiosoma; spermatheca not discerned	similar to *O.ussuriensis*; Bregetova 1977a
***dililoensis***		dense scale-like reticul. post., smooth or scattered reticul. ant.	reticulate	0	soft cut.	broad idiosoma	
***holaspis***	only HOLOG + METAP (partly to completely) fused	reticulate? (Oudemans’ text says “all shields with large scales”)	?	?	soft cut.?	elongate idiosoma	Oudemans (1903: 11) provided a more complete description than Oudemans (1902b); partly illustr. by Ryke (1962)
***mooiensis***		reticulate; reticul. sparser anteriorly	?	0–1	soft cut. or endop.?	elongate idiosoma; METAP rarely free (based on syn. *O gamagarensis*)	Marais and Loots 1972, Jordan and Loots 1987, Nemati et al. 2018 (notes on characters)
placentula*		essentially smooth (finely granulate) or faintly reticulate	lineate-reticulate	0	stern.	broad idiosoma; sternal shield wider than long, with concave margin; PERIT reaching past coxa IV; spermatheca not discerned	Sellnick 1940: 69, Ryke 1962, Bregetova and Koroleva 1964, Evans and Till 1966, Bai and Ma 2014
***platensis***		?	?	?	soft cut.?	peritreme short, reaching between coxae I–II; *ZV1* absent?	Ryke 1962 (partial illustration)
***rectagoni***		?	?	0	soft cut.?	*j1* seta elongate; broad idiosoma and HOLOG; PARAP truncate; *ZV3* apparently on HOLOG	Karg 1994 (male chelicera and spermatodactyl)
***sinensis***		?	?	?	soft cut.?		Ryke 1962 (partial illustration)
***ussuriensis***		polygonal reticul. scarcely evident (text)	lineate-reticulate	0?	stern.	spermatheca not discerned; only 2–3 deutosternal denticles / row	Bregetova 1977a
***bregetovae***	all shields (HOLOG, METAP, PERIT, PARAP) free	with (scale-like?) reticulation post.	?	0?	?	elongate idiosoma	similar to *O.tasmanicus* and *O.sitalensis*?
***expansus***		?	?	0?	soft cut.		
***nasri***		finely granulate?	lineate-reticulate?	0	soft cut.?	broad dorsal and sternal shields	similar to *O.obovatus*
***obovatus***		smooth?	?	?	soft cut.	broad idiosoma; *ZV1* absent?	
***paratasmanicus***		reticulate	?	0	soft cut.	elongate idiosoma; HOLOG rounded laterally	similar to *O.tasmanicus*; Ma 2015
***sitalaensis***		?	reticulate	1	soft cut.?	elongate idiosoma	
***tasmanicus***		lightly reticulate (Womersley’s text)	?	0	soft cut.	Tenorio (1982) indicates broader idiosomal shields than those in Womersley (1956)	Tenorio 1982 (photograph)

**Figure 1.**
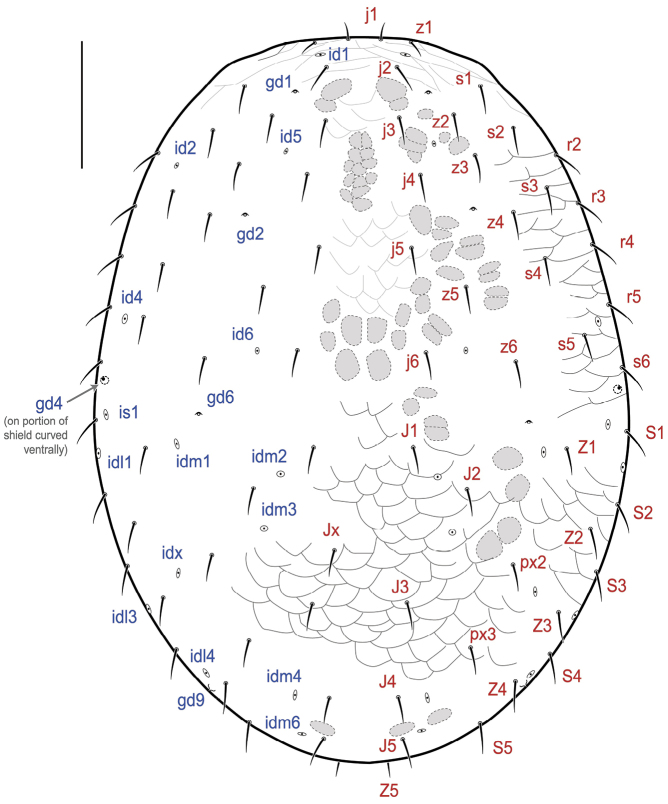
*Ololaelapsformidabilis*, adult female. Dorsal idiosoma. Note that setae *Z5* and poroid *idm5* are inserted on the ventral portion of the dorsal shield (see Fig. 2). Grey zones represent sigillae. Scale bar: 100 µm.

**Figure 2.**
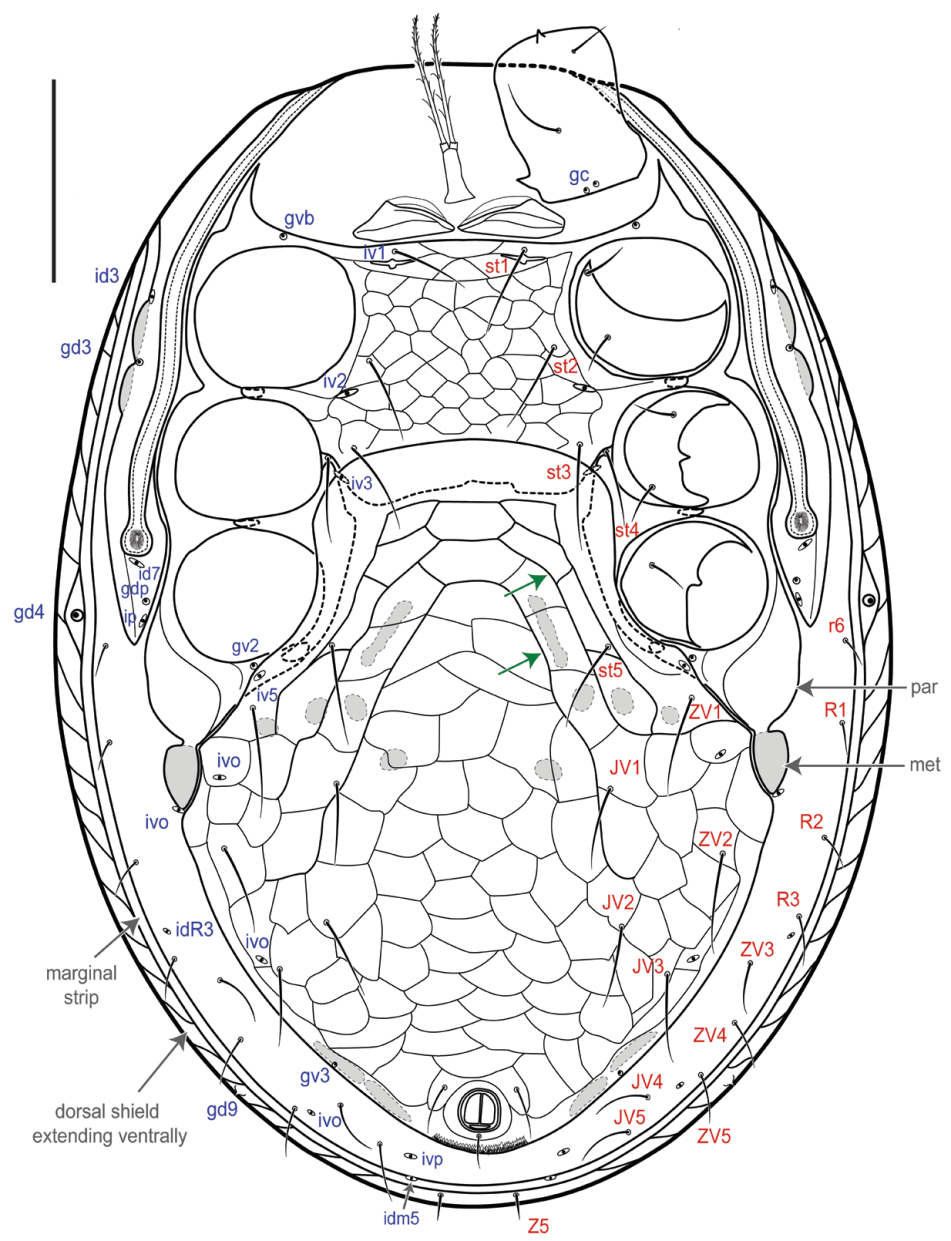
*Ololaelapsformidabilis*, adult female. Ventral idiosoma. Green arrows show the two inverted V-shaped ridges; other arrows indicate parapodal (“par”) and metapodal (“met”) plates. Scale bar: 100 µm.

? indicates unknown or uncertain data. **^1^**”Shields” include: HOLOG – hologastric, METAP – metapodal, PARAP – parapodal, PERIT – peritrematal; other acronyms or abbreviations: FD – fixed digit; MD – movable digit; ant. – anteriorly; post. – posteriorly; reticul. – reticulate or reticulation; spermatod. – spermatodactyl; spermath. – spermatheca. **^2^**Epipleura: portions of the dorsal shield that extend ventrolaterally (see Bregetova and Koroleva 1964); “lineate-reticulate” emphasizes that cells of the reticulation are stretched out so that they appear mostly as (parallel) lines (also parallel to the shield margin) instead of the typical scale-like (e.g., Fig. 1) or polygonal reticulation (Fig. 2, sternal shield) (“reticulate”). **^3^**Seta *st4* inserted on sternal shield (“stern.”), soft cuticle (“soft cut.”) or endopodal plate (“endop.”). **^4^**Bregetova and Koroleva’s (1964) text (for female) and illustrations (figs 17, 19: female and male, respectively) indicate that *O.sellnicki*’s epipleura are smooth, but Evans and Till’s (1966) illustration of the male shows epipleura with reticulation posteriorly.

### Remarks on the genus

We herein recognize 26 valid species names in the genus *Ololaelaps*, and at least four synonyms (Table 1). The majority of species need redescription, including four species that are nearly entirely unknown morphologically (*O.hemisphaera*, *holaspis*, *platensis*, *sinensis*). While some species are relatively well understood (e.g., *O.formidabilis*, *placentula*, *sellnicki*, *ussuriensis*, *wangi*), they nevertheless require additional study to elucidate intraspecific variability, in turn to better distinguish them from close relatives (Table 2). Table 2 presents some of the available diagnostic features of species, which are few. Indeed, identification of most species is problematic; our attempt to prepare a useful key to species was unsuccessful, due to the limited set of reliable diagnostic characters for most species. Other characters not presented in Table 2 may become useful (see Discussion), but intraspecific variability and their diagnostic potential remain to be determined. The case of *O.mooiensis*, a senior synonym of *O.gamagarensis* as established by Nemati et al. (2018), is a good example of intraspecific variation of characters, including the degree of fusion of the metapodal platelet – free to completely fused – with the hologastric shield, and the length of sternal setae. These two characters were apparent differences between *O.mooiensis* and *O.gamararensis* (Jordaan and Loots 1987) but now appear as mere variation along a range within a single species (A Nemati pers. comm.). Other names in Table 2 may represent synonyms.

The identity of *Iphishaemisphaericus* (Koch 1839b) is complicated. The species is placed by some authors in *Ololaelaps* (Laelapidae) and in *Stylochirus* (Ologamasidae) by others (Table 3). Berlese (1914) redescribed the species as Gamasiphis (Periphis) haemisphaericus (Koch) based on non-type specimens that he collected from Italy. Following Berlese’s concept, Sellnick (1958) and Vitzthum (1943) mention *Periphishaemisphaericus* (Koch), and Lee (1970) redescribed the species as *Stylochirus* (= *Periphis*) *haemisphaericus* using female specimens from Italy that Berlese (1914) himself had studied for his description. *Stylochirushaemisphaericus* (Koch) is listed in the catalogue of Ologamasidae by Castilho et al. (2016).

**Table 3.** List of species that have been previously considered in *Ololaelaps* (as genus or subgenus), but herein excluded or considered dubious species (nomina dubia).

Species	Original genus	Current genus	Key sources for current placement	Sources placing it in *Ololaelaps*	Additional notes	Type locality
***coleoptratus*** Berlese, 1888: 198	*Hypoaspis*	*Hydrogamasellus* (Ologamasidae)	Castilho et al. 2016	Berlese 1904: 261	Lee (1970: 113) redescribed the species based on types	ARGENTINA: Buenos Aires
***festivus*** Koch, 1839b: 8	*Zercon*	nomen dubium	–	Oudemans (1936: 216); he considered *Z.festivus* may be the deutonymph of *Iphishaemisphaericus* Koch, which he considered in turn as a syn. of *O.placentula*; Turk (1953: 12) accepted this syn., with *Hyletastesfestivus* (Koch) (Laelaptidae) as the valid name	See also Koch (1842: 91), Sellnick (1940: 68); the concept of the genus *Hyletastes* is not clear (see notes for *Iphisglobulus* below)	GERMANY: Neumarkt
***germanicus*** Karg, 1965: 277	Ololaelaps (Cypholaelaps)	*Pseudoparasitus* (Laelapidae)	Karg 1971, 1993a; Bregetova 1977a	Karg 1965		GERMANY: Zörbig
***globulus*** Koch, 1839b: 17	*Iphis*	nomen dubium	–	Oudemans (1902a: 289) considered *I.globulus* (as *Hypoaspis*) as syn. of *O.placentula*	Vitzthum (1943: 766) placed *Iphisglobulus* as the type species for *Hyletastes* (see also Oudemans 1936: 216, 218, 221)	GERMANY: Regensburg
***haemisphaericus*** Koch, 1839b: 16	*Iphis*	nomen dubium: either *Stylochirus* (Ologamasidae) or *Ololaelaps* (as syn. of *O.sellnicki*; see Table 1 and main text)	Castilho et al. 2016 (as *Stylochirus*)	Sellnick (1940) and subsequent authors (e.g., Willmann 1949, 1952, Halašková and Kunst 1961)	Lee (1970: 194) redescribed the species based on specimens identified by Berlese (1914: 142); Koch’s types seem to never have been re-examined	GERMANY
***haemisphaericus*** Berlese, 1916a: 166	Ololaelaps (Cypholaelaps)	nomen dubium; note that *Cypholaelapssemiglobulus*Vitzthum (1935) was considered similar to *Cypho.haemisphaericus* (Berlese)	–	Berlese (1916a), Vitzthum (1943: 763), as O. (Cypholaelaps); Karg (1965: 271), using a concept of O. (Cypholaelaps) for species now in *Pseudoparasitus*; Karg (1971) and Karg (1993a) mention it (wrongly) as syn. of Pseudoparasitus (O.) sellnicki	Berlese’s (1916a) species description incl. an “anal shield obtriangular”, in accord with description of subgenus O. (Cypholaelaps) (Berlese 1916a), which incl. an anal shield separate from (though contiguous to) a genitiventral shield	ARGENTINA: La Plata
***holostaspoides*** Canestrini, 1884: 700	*Laelaps*	*Sessiluncus* (Ologamasidae)	Bregetova 1977b; Castilho et al. 2016; see also Bregetova and Koroleva (1964)	Berlese 1904: 260; Oudemans (1902b, 1903) compares *holostaspoides* Can. with *holaspis* Oud. (as *Hypoaspis* spp.)	Unclear if types have been examined, but Canestrini (1884) indicates there are 3 shields ventrally, incl. an intermediate, semicircular shield (probably epigynal), which excludes it from *Ololaelaps*	ITALY: Messina
***inornatus*** Johnston, 1849: 305	*Eumaeus*	nomen dubium	–	considered a sen. syn. of *Ololaelapsconfinis* in Turk (1953: 12), within genus *Hyletastes*	See notes for *Z.festivus* above, and Oudemans (1936: 222)	UK (Scotland): Berwickshire
***pergibbus*** Berlese (in Castagnoli and Pegazzano 1985: 316)	*Ololaelaps*?	species name not available	–	Castagnoli and Pegazzano 1985	The species name was not published (see Castagnoli and Pegazzano 1985), therefore it is not available (ICZN article 11.1)	CHINA

Meanwhile, Sellnick (1940) redescribed the species as *Ololaelapshaemisphaericus* (Koch). His interpretation of *haemisphaericus* as an *Ololaelaps* species has been followed by some authors (Haarlov 1943, Franz and Beir 1948, Willmann 1949, 1950, 1952, Piryanik 1962, Reitblat 1963) until Bregetova and Koroleva (1964) proposed *O.sellnicki* as a nom. nov. for *O.haemisphaericus* (Koch 1839b). Bregetova and Koroleva (1964) argued that using a new name was better than using the confusing name *haemisphaericus*, which was also applied to other species in at least one other family. Before Sellnick (1940), Oudemans (1906, 1929, 1936) mentioned *Iphishaemisphaericus* as conspecific either with *O.placentula* or with *O.venetus*. Oudemans (1936: 217) stated that Berlese erroneously identified a different species as “*Periphishaemisphaericus*” (certainly referring to Berlese 1914).

There is no indication that anyone examined Koch’s types of *haemisphaericus*, and the types of most species described by Koch are presumably lost. Therefore, it may be impossible to confirm with certainty whether Koch’s species is *Stylochirus* or *Ololaelaps*. Resolving this dual identity of *Iphishaemisphaericus* (Koch 1839b) will require submitting a case to the International Commission of Zoological Nomenclature. Because the name *Ololaelapssellnicki* Bregetova and Koroleva is frequently used, and the name *haemisphaericus* Koch has been more recently applied in the sense of an ologamasid and not as an *Ololaelaps* species, the best approach may be to designate (1) a neotype for *Stylochirushaemisphaericus* (Koch 1839b) and (2) a lectotype for *Ololaelapssellnicki*Bregetova and Koroleva (1964) in order to maintain the prevailing concepts of these names. Note that Bregetova and Koroleva (1964) had not designated a type for *O.sellnicki* since they treated *sellnicki* as a replacement name for *haemisphaericus*, but the specimens they studied can be considered as syntypes.

Hennessey and Farrier (1988) synonymized *O.venetus* (Berlese 1903), a Palearctic species (and the type species of the genus), with *O.placidus* (Banks 1895), a species otherwise previously restricted to the Nearctic region. However, despite Hennessey and Farrier’s (1988) analysis, we refrain from accepting this synonymy because we consider that these two species (or populations) from North America and Eurasia are not known in sufficient details yet (see further explanations in the Discussion). Nonetheless, we accept for now the synonymy of *O.venetus* and *O.halaskovae* (the latter is also Palearctic), which was originally proposed by Evans and Till (1966) and also accepted by Bregetova (1977a). However, Evans and Till (1966) did not specify what specimens they used for their redescriptions of *O.venetus* and *O.placentula* and whether they examined Berlese’s types.

Some species names once considered as *Ololaelaps* are herein excluded from the genus, based on the interpretation of the original description or more recent publications (Table 3): the two ologamasids *Hydrogamasuscoleoptratus* and *Sessiluncusholostaspoides*, the laelapid *Pseudoparasitusgermanicus*, and the nomen dubium Ololaelaps (Cypholaelaps) haemisphaericus Berlese (not Koch). The type of the latter should be re-examined. From our current understanding, other species with doubtful identity cannot be excluded from *Ololaelaps* with certainty: *Zerconfestivus*, *Iphisglobulus* and *Eumaeusinornatus* (Table 3). Unfortunately, the types of those species may be lost. Note that *Ololaelaps* is distinct from ‘*Oolaelaps*’ which usually refers to species now placed in *Holostaspis* (Laelapidae) (Keum et al. 2017).

Although Evans and Till (1966) treated the genus *Ololaelaps* as feminine (indicated by *O.venetus*), Berlese (1904) originally treated it as masculine, indicated by two species that he originally included in the genus which had names in adjectival forms with clear masculine ending: *O.venetus* and *O.coleoptratus*. We herein follow Berlese and treat *Ololaelaps* as masculine for the following reason. As per Article 30.1.1 of the ICZN, “a genus-group name that is or ends in a Latin word takes the gender given for that word in standard Latin dictionaries”. The name *Ololaelaps*, as created by Berlese, probably stands for ‘*holo*’, ancient Greek for ‘complete’, putatively referring to the nearly completely sclerotized idiosoma, or opisthogaster, of the mites he included in the genus at the time; and ‘*laelaps*’, borrowed from the generic name *Laelaps*, first used by Koch (1836). Like Berlese (e.g., *Laelapsspiniferus* Berl., *L.myrmecophilus* Berl.), Koch appears to have treated *Laelaps* as masculine (as in *L.festinus*Koch 1839a). In Latin dictionaries (e.g., Lewis and Short 1879), *Laelaps* is masculine and refers to the Greek mythological dog of that name. Koch’s choice itself was almost certainly for that mythological hound which was known to never fail to catch its prey. ‘Laelaps’ was originally borrowed from Greek and means ‘hurricane’. Treating *Ololaelaps* as masculine results in the change of a single species name from its original ending: *O.obovata* to *O.obovatus*. Note that some species names are feminine, such as *placentula* (= little cake) and *hemisphaera* (= hemisphere), but these are nouns in apposition and have therefore invariable spellings, irrespective of the gender of the genus.

#### Ololaelaps formidabilis

Taxon classificationAnimaliaMesostigmataLaelapidae

Berlese, 1913

Figs 1 , 2 , 3 , 4 , 5 , 6 , 7 , 8

##### Diagnosis.

Dorsal shield broad, length/width ratio ~1.3–1.4, lightly reticulate, bearing 39 pairs of simple setae, including *px2–3*, plus one unpaired seta *Jx* (sometimes absent); all setae short (21–27; j1, z1, J5 shorter); shield with gland opening gd4 conspicuous, on shield margin; epipleura narrow, strongly reticulate. Female sternal shield as long as wide (length/width ratio 0.96–1.02), bearing setae st1–st3; seta st4 and poroid iv3 on soft cuticle. Hologastric shield with two inverted V-like ridges, and strongly reticulate; cells scale-like in region anterior to anus, bearing seta st5 and five pairs of preanal setae. Soft opisthogastric cuticle laterad of shield with nine pairs of setae. Peritrematal shield free posteriorly, reaching level of coxa IV posterior margin. Metapodal shield suboval, narrowly fused to parapodal shield (and contiguous with hologastric shield) in female. Deutosternal groove with 3–5 denticles per row. Spermatodactyl prominent, 1.8× as long as movable digit.

##### Female

**Figure 3.**
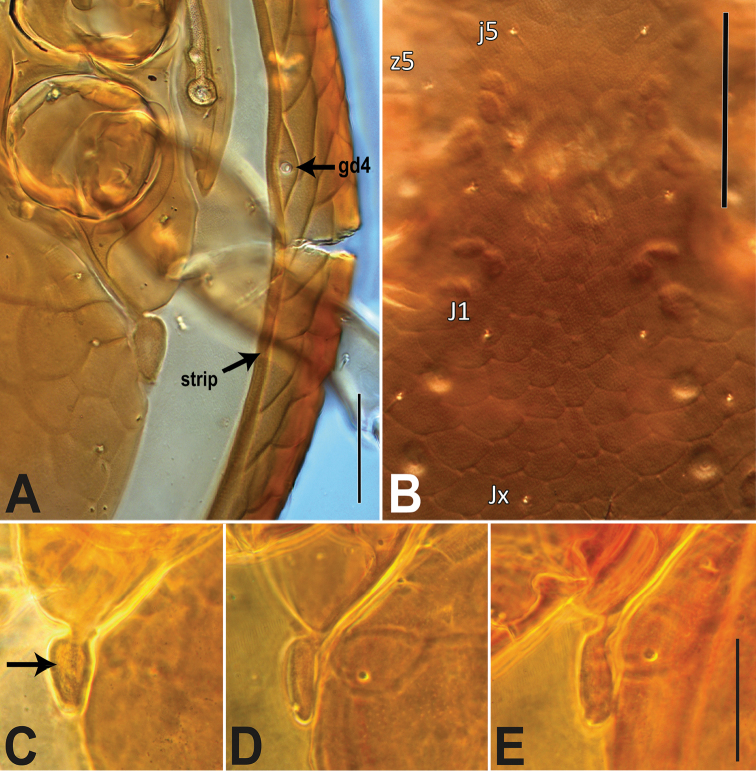
*Ololaelapsformidabilis*, adult female. **A** ventrolateral region of idiosoma, showing the well-reticulated epipleuron (ventrolateral portion of dorsal shield), gland opening gd4, and the dorsal shield’s marginal strip (“strip”); note that the epipleuron appears broader than in live specimen, because the specimen was squashed on the slide, as indicated by the broken dorsal shield **B** central region of the dorsal shield, showing the light reticulation of the opisthonotal area (near J1, Jx) and even lighter reticulation of the podonotal area (see between setae j5) **C–E** metapodal platelet (arrow), variously fused to the parapodal plate and contiguous with the hologastric shield. Scale bars: 50 µm (**A**); 100 µm (**B**); 50 µm (**C–E**).

**Figure 4.**
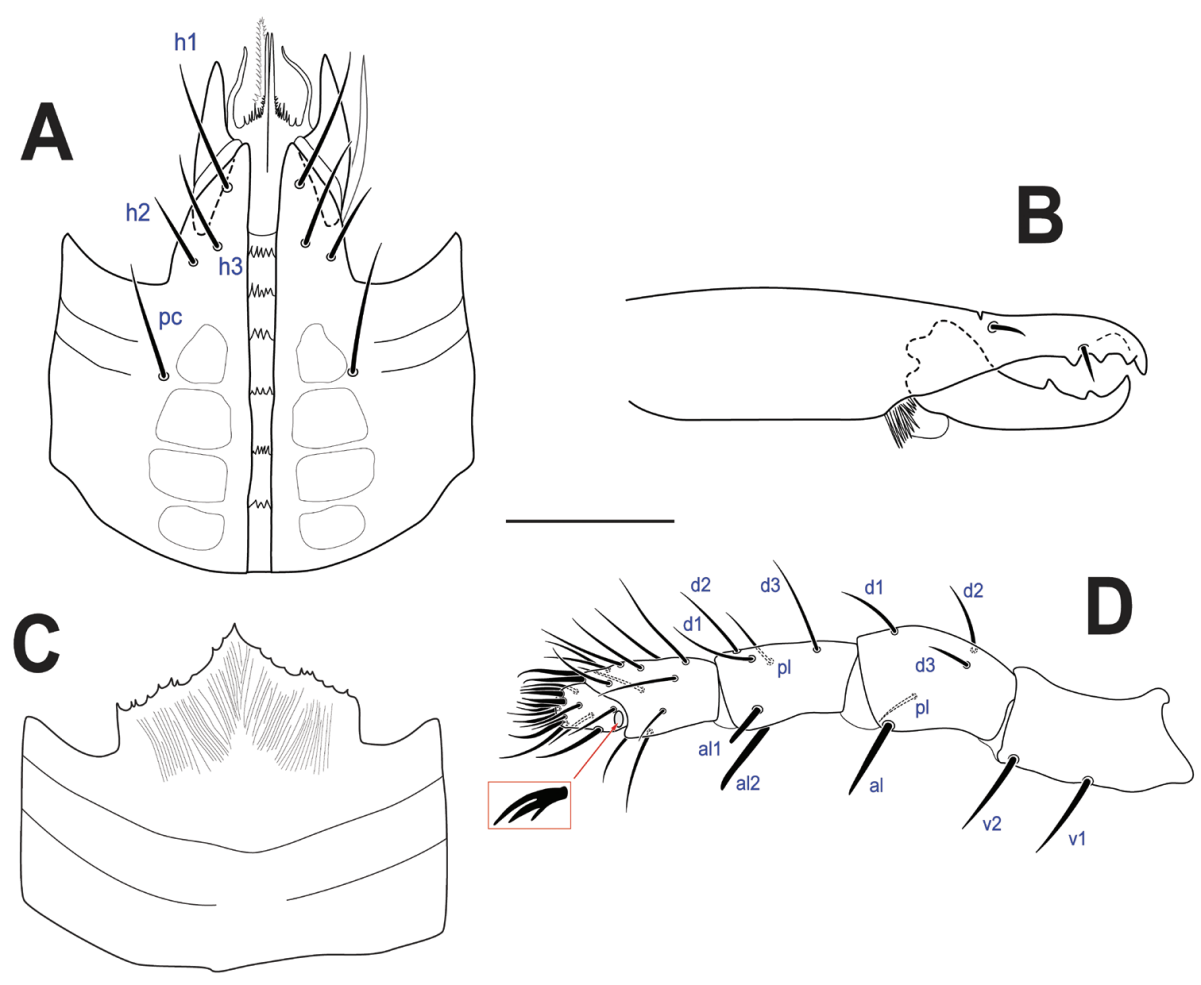
*Ololaelapsformidabilis*, adult female **A** subcapitulum **B** chelicera (antiaxial view) **C** gnathotectum **D** palp, with inset showing palp apotele. Scale bar: 50 µm.

**Figure 5.**
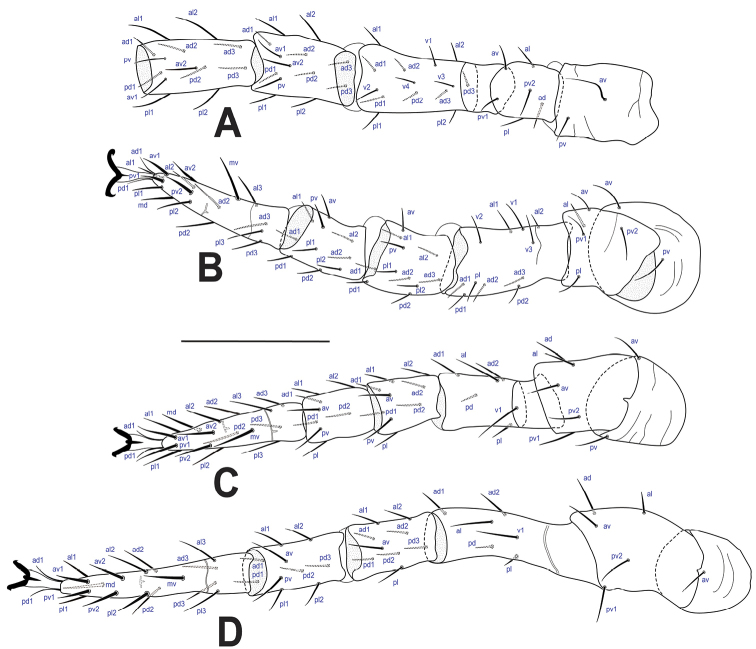
*Ololaelapsformidabilis*, adult female **A–D** legs I–IV, respectively. Scale bar: 100 µm.

(Figs 1–5) (n = 3). **Description. *Idiosomal dorsum*** (Figs 1, 2, 3B). **Dorsal shield** 567–607 long, 410–440 wide (near level of seta S1), covering all dorsal idiosoma, oval-shaped, dome-like, strongly sclerotized and slightly covering ventrolateral margins (epipleura), with a light reticulation on most areas of shield, more conspicuous in opisthonotal region (as shown in region of J3 vs region between j5 and z6) and epipleura strongly reticulate; region anterior to setae j2–s1 with conspicuous, transverse lineae; shield with a delineated marginal strip along its edge (Figs 2, 3A). Shield with 39 pairs of simple setae: j1–j6, z1–z6, s1–s6, r2–r5 on podonotal region, J1–J5, Z1–Z5, S1–S5, px2–3 on opisthonotal region, and usually one unpaired seta Jx (absent in one of three females) inserted on right side (one female) or left side (another female) of shield’s median axis. All dorsal setae slender, relatively short (21–27), with j1, z1 and Z5 shorter (11–15); distance between J5 setae 62–66, distance between Z5 setae 40–46. Dorsal shield with 21 pairs of pore-like structures, including five pairs of gland openings (gd1, gd2, gd4, gd6, gd9) and 16 pairs of poroids; gd4 large, on lateral shield margin (discernible ventrally), posterolaterad of s6 (and level with mid-coxa IV), surrounded by a curved linea (Figs 2, 3A).

***Idiosomal venter*** (Figs 2; 3A, C–E). **Tritosternum** with columnar base and a pair of pilose laciniae. **Presternal area** with a pair of well-sclerotized presternal platelets, wedge-shaped, with transverse lineae; region anteromesal to platelets poorly sclerotized, lineate and granulate. **Sternal shield** 118–125 long, 122–125 wide (at level of setae *st2*), strongly reticulate, smooth in posterior fifth where overlapped by hologastric shield, with inconspicuous punctae; anterior shield margin straight and posterior shield margin slightly concave, bearing three pairs of simple, slender setae, st1–3 (44–65), and slit-like poroids iv1–2; st1–st1 distance 65–70, and st1–st3 distance 93–98; st4 (45–48) and iv3 on soft cuticle (which may overlap endopodal plate), near posterolateral margin of sternal shield, mesal to coxa III. **Endopodal shield** besides coxa III–IV large, free, narrowly abutting sternal shield, slightly overlapped by hologastric and exopodal shields. **Exopodal shield** surrounding acetabula II–IV narrowly fused with sternal shield (via endopodal element) anteriorly between coxae I–II, posteriorly fused with well-developed parapodal element. **Peritrematal shield** fused anteriorly to dorsal shield at level between coxae I–II, posteriorly free, not extending beyond posterior margin of coxa IV, bearing three pairs of poroids (id3, id7, ip) and two pairs of gland pores (gd3, gdp); peritreme extending anteriorly beyond coxa I, near level of seta z1. **Hologastric shield** strongly reticulate, 359–366 long, 289–301 wide; one or two discernible inverted-V ridges in anterior half of shield (the anterior ridge may be less evident in some individuals); cells more compressed, scale-like (and narrow, transversally elongate) in region directly anterior to anal opening; shield with inconspicuous punctae; bearing six pairs of slender setae, st5, JV1–3, ZV1–2 of subequal length (37–53), three pairs of poroids, including paragenital poroids iv5; st5–st5 distance 130–138; insertion of paranal setae (24–30) aligned with anterior margin of anal opening, postanal seta shorter (12–19); gland opening gv3 on posterolateral shield margins, at level slightly anterior to paranals; cribrum with 2–3 rows of spicules. Soft opisthogastric cuticle with nine pairs of setae, r6, R1–2 (15–22), R3, ZV3–5, JV4–5 (19–35), four poroids, including one (ivo) at posterior edge of metapodal platelet, and another (idR3; = Rp) near seta R3. **Metapodal** element oval-shaped, narrowly fused to parapodal-exopodal shield (Fig. 3A, C–E) and contiguous with hologastric shield (may also appear narrowly, inconspicuously fused to hologastric shield in some individuals).

***Gnathosoma*** (Fig. 4). **Subcapitulum** (Fig. 4A): corniculi horn-like (45–51); internal malae with two pairs of long projections, slightly longer than corniculi, median pair fringed at its base; labrum acuminate, slightly longer than internal malae; hypostomal and capitular setae smooth, h1, h3, pc (27–44), h2 shorter (20–24); deutosternal groove with five (1 female) or six rows (2 females) of denticles, progressively broader from posterior to anterior, each with 3–5 denticles. **Cheliceral** (Fig. 4B) fixed digit (63–68) with a subapical, offset tooth, followed by two moderately large teeth and setiform pilus dentilis, movable digit with two similarly sized teeth; simple dorsal seta. **Gnathotectum** (Fig. 4C) with anterior margin subtriangular, irregularly and lightly serrate. **Palp** (Fig. 4D) with normal chaetotaxy for Laelapidae (sensu Evans and Till 1965), with 2-5-6-14-15 setae on trochanter-femur-genu-tibia-tarsus; palptrochanter setae v1 and v2 thickened; palpfemur al thickened, blunt apically, palpgenu al1, al2 thickened, spatulate (flat and rounded) apically; palp-tarsal claw with three tines, third (proximal) one smaller.

***Legs*** (Fig. 5). Chaetotaxy normal for Laelapidae (sensu Evans and Till 1966). Leg II slightly thicker than other legs. Lengths of legs: I 471–485, II 360–381, III 342–360, IV 470–485. All legs with ambulacral stalk, claws and pulvillus; entire ambulacrum I (26–28), including claw I (8–10), slightly shorter than ambulacra II–IV (31–39) and claws II–IV (12–15), respectively. Most setae slender and of moderate length, except a few shorter and/or thickened setae: femur II with al2 short; femur III–IV with pd and pl 2–3 times shorter than v1 and al; tarsi II–IV with av1–2, pv1–2, mv, md thickened, and md, al1–2, pl1–2 slightly thickened, pl2 thickened on tarsus IV.

***Spermatheca.*** Not discerned.

##### Male

**Figure 6.**
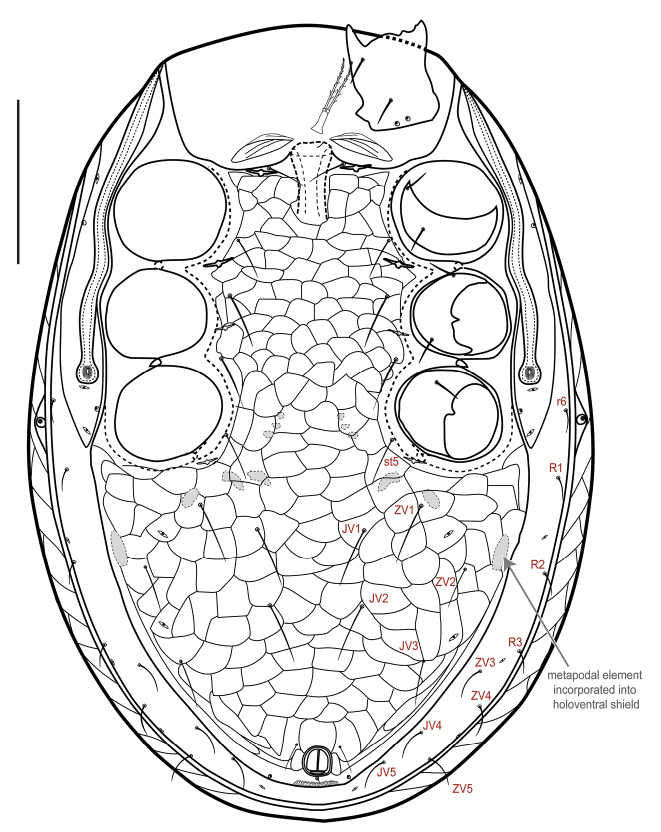
*Ololaelapsformidabilis*, adult male. Ventral idiosoma. Scale bar: 100 µm.

**Figure 7.**
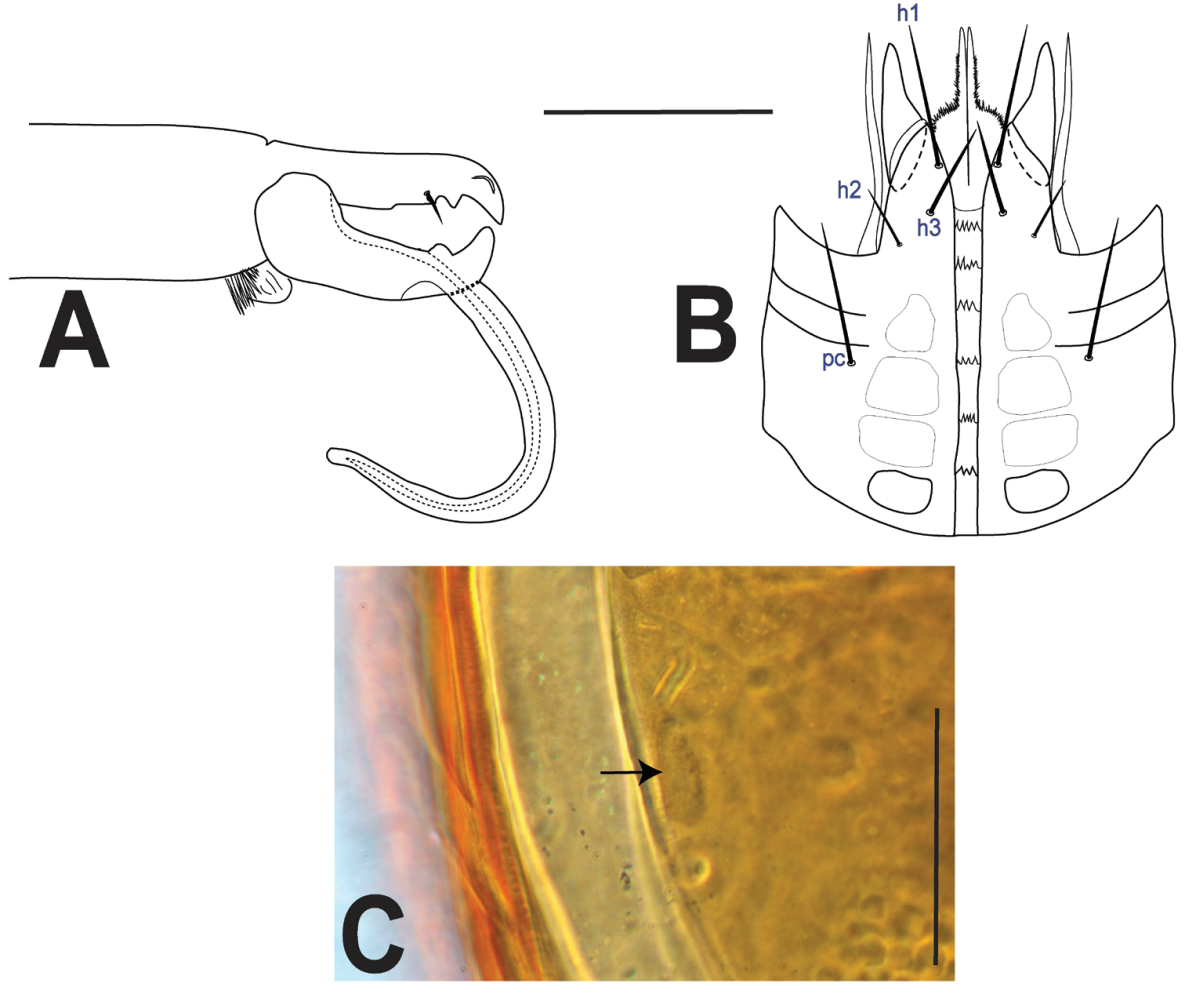
*Ololaelapsformidabilis*, adult male **A** chelicera **B** subcapitulum **C** posterolateral region of idiosoma, showing metapodal element (arrow) integrated in the holoventral shield. Scale bars: 50 µm.

(Figs 6–7) (n = 1) **Description. *Idiosomal dorsum***. **Dorsal shield** 493 long, 382 wide (at level of setae *S1*), as female: covering all dorsal idiosoma, oval-shaped, dome-like and slightly covering ventral surface. Poroidotaxy, adenotaxy, chaetotaxy and ornamentation essentially identical to those of female; setae slightly shorter.

***Idiosomal venter*** (Fig. 6). Similar to female except the following: **holoventral shield** 380 long, 106 wide at level of st2, 267 wide at level of ZV1, strongly reticulate; shield bearing 10 pairs of simple, slender setae (st1–5, JV1–3, ZV1–2) in addition to circumanal setae. **Exopodal** shield fused with holoventral shield posteriorly to coxa IV, and extending anteriorly to level of mid-coxa I. **Metapodal** element (sigillum) incorporated into holoventral shield (see arrows, Figs 6, 7C).

***Gnathosoma*** (Fig. 7). As female, except: **subcapitulum** (Fig. 7B): internal malae without the pair of lateral projections, and median projections more fimbriate than in female; deutosternal rows each with 3–5 denticles. **Cheliceral** (Fig. 7A) fixed digit with one tooth; movable digit with one tooth, subapically bearing an elongate spermatodactyl (102), broadly curved, slightly bent apically, with straight (i.e., not sinuous) duct.

***Legs.***Chaetotaxy and setae thickness similar to that of female. Lengths of legs: I 406–415, II 301–310, III 295–305, IV 380–395.

##### Material and depository.

INDONESIA, Sumatra • 1♀, Harapan rainforest, litter from rubber tree plantation, research site HR4b, 01°48'18"S, 103°15'52"E, 71 m a.s.l. (LIPI; internal project ID macrolitterHR4b13_MESOS1_1) • 1♀, same data as preceding (CNC1098357; internal project ID macrolitterHR4b13_MESOS1_2) • 1♀ (with an egg), Bukit Duabelas rainforest, litter in rubber tree plantation, research site BR4b, 02°04'36"S, 102°46'22"E, 51 m a.s.l. (SMNG-ARA-13/59952; internal project ID macrolitterBR4b13_MESOS1_1) • 1♂, same data as preceding (LIPI; internal project ID macrolitterBR4b13_MESOS1_2). All specimens collected on 15.11.2013 by B. Klarner. Additional photos of the species are digitally deposited at ecotaxonomy.org.

##### Remarks.

**Figure 8.**
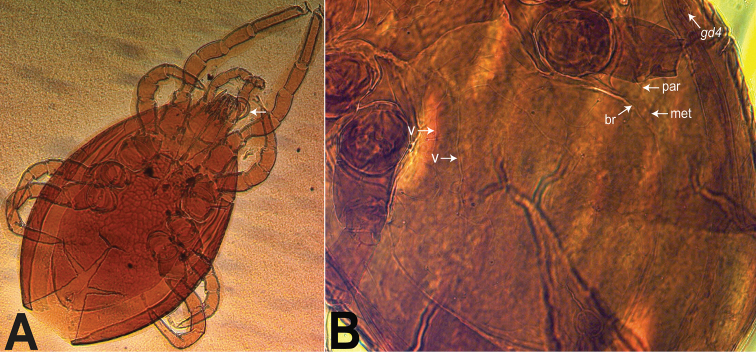
*Ololaelapsformidabilis***A** male holotype (slide 145/29): ventral view, with arrow pointing at spermatodactyl **B** female paratype (slide 145/30): region of hologastric shield, showing two inverted V-shaped ridges (v), and the ‘bridge’ (br) connecting parapodal (par) and metapodal (met) plates. Photographs courtesy of Roberto Nannelli.

Our discovery of *Ololaelapsformidabilis* in Sumatra appears to be the second record of the species in Indonesia, the first corresponding to the original description by Berlese from Java specimens. It is unique among described species of *Ololaelaps* in having its metapodal platelet fused to the parapodal plate and free from the peritrematal and hologastric shields. Note, however, that the metapodal platelet is tightly contiguous with the hologastric shield and that in some specimens, at some focal depth, it may even appear narrowly fused with it (Fig. 3A). The metapodal and parapodal plates are fused by a short to elongate connecting ‘bridge’ (Fig. 3A, C–E). Photos shared by Roberto Nannelli, who examined types at the Berlese Collection in Firenze, confirm that at least one female paratype of *O.formidabilis* has such attribute, although the connecting bridge between the metapodal and the parapodal plates seem slightly broader (Fig. 8B; although not perfectly clear) than for the three females from Sumatra (Fig. 3A, C–E). Berlese’s (1913) original description (fig. 51, plate V) shows a fusion (‘bridge’) that is as broad as the width of the metapodal platelet. We consider that the difference between the paratype and our specimen represents intraspecific variation. In addition, *O.formidabilis* has two inverted-V-shaped ridges on the anterior half of its hologastric shield (see arrows, Fig. 1). The posteriormost ridge, shaped more narrowly, is more conspicuous than the anterior one (which is almost U-shaped). The female paratype photographed shows similar ridges (Fig. 8B). Although at least two undescribed species have similar inverted V or U ridges, the shapes of the ridges in these species are distinct from those of *O.formidabilis*.

The male holotype of *O.formidabilis* (Castagnoli and Pegazzano 1985: 151) is also similar to that of the new material, including for its spermatodactyl, which has a similar thickness and length (see arrow, Fig. 8A).

Ryke (1962) redescribed *O.formidabilis*, via a species key and a single illustration, of the idiosomal venter, which clearly represents another species, distinct from *O.formidabilis* described by Berlese (1913) and examined by us. The most distinctive character in Ryke’s illustration (his fig. 6) is the metapodal platelet, broadly protruding from its fusion with the hologastric shield, but free from the parapodal shield, in contrast to *O.formidabilis* sensu stricto. Such partial fusion of the metapodal-hologastric shield is similar to nine other species in the genus (*O.caucasicus*, etc., Table 2). Other information included in the key of Ryke (1962), such as idiosomal dimensions and geographic origin (Java), corresponds to those of *O.formidabilis*, but were probably simply taken from Berlese’s publication (except that Ryke indicated “length 550 μ” instead of 540 μ as written in Berlese (1913)). In the introduction, Ryke (1962) thanked G.O. Evans for “putting […] the figures of the type specimens in the Berlese Collection at his disposal”. From this, we could interpret that during a visit of the Berlese Collection in Firenze, Italy, Evans examined types and illustrated them, and later on, lent these illustrations to Ryke. We attempted to retrieve putative illustrations by Evans, or Ryke, but without success. It is possible that a mistake occurred at some point and that Ryke’s (1962) illustration is that of a type or voucher specimen representing another species. At present, diagnostic characters included in Ryke (1962) are too limited to determine the correct name of that species (if it has one). Re-examination of *Ololaelaps* specimens in the Berlese Collection might help resolve this.

## Discussion

### Features of the genus

At present, *Ololaelaps* appears as a relatively well-defined genus, characterized by a unique combination of characters, many of which, individually, are shared with other genera of Laelapidae, especially hypoaspidines. The most unique feature of *Ololaelaps* is the female genital shield hyperdeveloped posteriorly and fused with the anal shield to occupy most of the opisthogaster and capture 3–5 pairs of setae in addition to st5 and circumanals. The genital shield is also expanded in several other genera (e.g., *Laelaspis*, *Laelaspisella*, *Pseudoparasitus*, *Pogonolaelaps*; Evans and Till 1966, Hunter 1966, Joharchi et al. 2016, Nemati and Gwiazdowicz 2016) but it is never fused to the anal shield like in *Ololaelaps*, except in *Oloopticus* (Karg 1978). *Oloopticus* is distinguished from *Ololaelaps* at least by the sternal shield coalesced anteriorly with presternal platelets and posteriorly with endopodal plates, and by the modification of setae st4 into sensory ‘pits’. Karg and Schorlemmer (2013) suggested that *Ololaelaps* and *Oloopticus* are closely related genera, based on the hypothesis that they apomorphically share a hologastric (genitiventrianal) shield (Karg 2000). However, this character state could have evolved independently in these two genera, which otherwise appear phylogenetically distant. The fusion of epigynal + ventral + anal shields also occurs in members of Eviphidoidea, such as *Holaspulus*, some *Holaspina* (Parholaspididae; Halliday 1995, Nawar and El-Sherif 1995) and *Indutolaelaps* (a genus similar to *Holaspina*; Leptolaelapidae; Karg 1997).

The hemispherical nature of the idiosoma of several species of *Ololaelaps* is also distinctive. However, this attribute may have led to misidentifications or misclassifications in the past, as some species in other families, especially Ologamasidae, have a similarly glossy, dome-shaped dorsal shield (see Table 3). That would explain in part the apparent dual identity of *Iphishaemisphaericus*, associated with two phylogenetically distinct genera, *Stylochirus* (Ologamasidae) and *Ololaelaps* (see Remarks for the genus above). Unfortunately, Koch (1839b) illustrated only the dorsal aspect of that mite. Another similarity is that ologamasids also tend to be strongly sclerotized ventrally, and that may have added to the confusion. Old species names of uncertain identity (Table 3: *Zerconfestivus*, *Iphisglobulus*, *Eumaeusinornatus*) may have been historically associated with (valid) *Ololaelaps* species for similar reasons. Some Eviphididae also have subglobular, domed idiosomas (Mašán and Halliday 2010).

As explained in Kazemi and Beaulieu (2016), the recently described monotypic genus *Persicolaelaps* shares many features with *Ololaelaps*, notably the dome-like dorsal shield bearing attenuate setae, and well-developed exopodal strips that are fused anteriorly with the sternal shield’s anterolateral arms (via endopodal elements). Note that such (anterior) fusion of exopodal-sternal shields occurs in other laelapids (e.g., *Alloparasitusoblongus* (Halbert); Evans and Till 1966). A pair of sclerotized presternal platelets, a three-tined palp-apotele, and well-developed parapodal plates characterize *Ololaelaps* as well as species of other hypoaspidine genera, especially of *Pseudoparasitus* and *Gymnolaelaps* (Hunter 1966, Joharchi et al. 2011, Nemati and Gwiazdowicz 2016). In addition to both having a reduced third (proximal) tine in their palp apotele (in contrast to a well-developed proximal tine in at least some *Gymnolaelaps*), *Pseudoparasitus* and *Ololaelaps* are also similar in having setae JV1 and JV2 inserted on the genital shield, but remotely from the lateral margins (vs on or near the shield margins in *Gymnolaelaps* or *Laelaspis*) (Joharchi et al. 2011). A three-tined palp-apotele was used as one of the main characters defining previous concepts of the family Neoparasitidae (Vitzthum 1943, Evans 1957, Mašán and Halliday 2014: 51) which was composed of various genera now scattered in at least four relatively distant families of Gamasina.

An additional set of features that further distinguish some *Ololaelaps* species from other laelapid genera is the various fusions of the peritrematal, parapodal, metapodal and hologastric shields. Even for groups with opisthogastric (i.e., genitiventral) shields such as *Laelaspis* and *Pseudoparasitus*, we are not aware of such fusion among shields. The peritrematal and parapodal shields, however, are coalesced in a few other laelapids, such as *Nidilaelapsannectans* (Womersley) (Shaw 2012).

The female of some *Ololaelaps* species have seta *st4* and poroid *iv3* on the sternal shield. This is rare in laelapids, although common within other groups, especially Rhodacaroidea. Seta *st4* is also born on the sternal shield (complex) in groups where the shield is fused posteriorly with endopodals (e.g., many ologamasids and pachylaelapids). However, in *Ololaelaps*, this feature seems associated with the anterolateral expansion of the genital shield, which leaves little soft cuticle available for the insertion of st4 and iv3. In other genera where the genital shield is more pronounced anteriorly, st4 has even disappeared (Kazemi and Beaulieu 2016). This ‘weaker’ seta, formed only during the deutonymphal stage, is also repressed in the adults or even the deutonymphs of other gamasines (Evans and Till 1965, Karg 2000, Lindquist 2003, Kazemi and Beaulieu 2016).

The males of *Ololaelaps* are not as distinctive as females, although they can be distinguished from those of most other laelapid genera by the degree of development of the holoventral shield posterolaterally and its fusion to parapodal-exopodal shields, and sometimes to the peritrematal shields. However, a similar ventral shield arrangement occurs in the males of other laelapids, for instance *N.annectans* (Shaw 2012) and *Pseudoparasitusmissouriensis* (Ewing) (as *P.austriacus* (Sellnick), Hunter 1966). The ventrolateral extensions of the dorsal shield is an additional feature facilitating genus diagnosis (occurring in both sexes of *Ololaelaps*), which is uncommon in Laelapidae, and perhaps otherwise limited to species of *Gymnolaelaps* (Evans and Till 1966, Joharchi and Halliday 2013). Also, the peritrematal shield is fused to the dorsal shield along most of its length in the male of some species (e.g., *O.ussuriensis*).

### Species-level delineation

While it may be easy to identify a given *Ololaelaps* mite to genus, it is more difficult to identify it to species. Examination of types, as well as a critical assessment of intraspecific variation based on additional specimens will be necessary to clarify species boundaries and uncover synonymies. In particular, the following characters should be scrutinized during species (re)descriptions.

The type of fusion between metapodal, peritrematal, parapodal, and hologastric plates appears as a useful starting point to initiate species identification, because it sorts species into broad groups, which are phylogenetically meaningful in some cases (Table 2). Bregetova and Koroleva (1964) implied, in their key, the existence of two species groups, which have been further defined by Evans and Till (1966) based on a limited number of species: the *venetus* and *placentula* groups. The *venetus* group represents a small cluster of similar species (*O.venetus*, *placidus*, *sellnicki*) that nonetheless need further study. It is herein defined by five characters, some of which are likely derived (apomorphic), relative to the ancestor of the genus (Table 2):

(1) Fused peritrematal-hologastric-parapodal shields by way of the metapodal platelet. Note that the parapodal plate ranges from clearly to ambiguously fused to, or merely tightly contiguous with, the metapodal ‘bridge’ (e.g., compare figs 74–76 in Hennessey and Farrier (1988), figs 17, 21 in Bregetova and Koroleva (1964), and figs 49–50 in Evans and Till (1966)). Regarding this character, *O.hemisphaera* (Berlese 1916b) appears similar to species of the *venetus* group because its parapodal plate, albeit free, is (nearly) contiguous with the peritrematal shield (or metapodal bridge) (based on Ryke 1962). The peritrematal shield is also fused to the hologastric shield in *O.interruptus* and *O.leptochelae*, but the parapodal plate is clearly free of the fusion. Note that the original illustration of *O.venetus* by Berlese (1889; as misidentified “*Laelapstumidulus* (Koch)”) shows both the peritrematal and parapodal plates free from the hologastric shield, which is discordant with all other descriptions of *O.venetus* (or syn. *O.halaskovae*; Table 2). However, the illustration of the male spermatodactyl (Berlese 1889) shows a sinuous groove, like that of *O.venetus* and related species.

(2) Spermathecal ducts (= tubuli annulati, Evans 1992) well sclerotized, conspicuous, and similarly shaped in *O.venetus*, *O.placidus* and *O.sellnicki*. The spermathecae were also illustrated for *O.translineatus* (Barilo 1991) and *O.mooiensis* (including the sacculus foemineus; Marais and Loots 1972, Jordaan and Loots 1987), but they seem distinct from those of the *venetus* group of species. Hennessey and Farrier (1988) synonymized *O.venetus* with *O.placidus* certainly in part based on their similarity in the shape of the spermathecal ducts. They have indeed similar ducts, but the variation that we have observed between females of *O.placidus*, and between *O.placidus* and one or more undescribed, closely related species suggests that the shapes of the spermathecal ducts may overlap between species. The distinction between the spermatheca of *O.sellnicki* vs *O.venetus* or *O.placidus* may also not be so straightforward, given that the short subapical appendage characteristic of the ducts of *O.venetus* and *O.placidus* is not always discernible, and also that the ducts of all three species can be seen as apically ‘closed’ and rounded, or open-ended (Bregetova and Koroleva 1964; Hennessey and Farrier 1988; FB, pers. obs.).

(3) Spermatodactyl with a sinuous duct, and a subapical hump or bend (Bregetova and Koroleva 1964; Evans and Till 1966; F.B. pers. obs. for *O.placidus*). In contrast, the males of *O.formidabilis*, *O.placentula*, *O.translineatus* and *O.ussuriensis* have spermatodactyls of various lengths with a straight duct and no hump subapically; the spermatodactyl of *O.rectagoni* also has a straight duct and is swollen subapically (Karg 1994).

(4) Dorsal shield with narrow, smooth epipleura (i.e., ventrolateral extensions of the dorsal shield) vs broad, lineate-reticulate epipleura of the *placentula* group. Other species may have narrow epipleura, smooth or reticulate but descriptions are often lacking in such details, in part because determining the extent of the epipleura is most readily done before slide-mounting of the specimen (Barilo 1991) or on slide-mounted specimens with unbroken dorsal shield.

(5) A fifth character associated with the *venetus* group is the insertion of setae *JV3* and *ZV2* off the hologastric shield in *O.sellnicki* and in some individuals of *O.venetus* and *O.placidus* (Table 2; Ryke (1962), Bregetova and Koroleva (1964); FB, pers. obs. for *O.placidus*). This contrasts with all other known species, described with JV3 and ZV2 on the hologastric shield. Two other deviations from normal are seen in the illustrations of *O.obovatus* (Womersley 1960) and *O.platensis* (in Ryke 1962), both lacking ZV1, and of *O.rectagoni* (Karg 1993b) having ZV3 inserted on the shield.

The *placentula* group was defined by four characters (three mentioned by Evans and Till (1966), a fourth one only by Bregetova and Koroleva (1964)), none of which are clearly apomorphic, considering their (albeit poorly known) distribution across species in the genus (Table 2):

(1) six other species have the metapodal platelet fused to the hologastric shield (and free from parapodal/peritrematal plates), making this type of fusion relatively common in the genus (Table 2);

(2) a poorly sclerotized (i.e., inconspicuous) spermatheca may characterize other species, given that it has been described in five species only (see above);

(3) at least two other species have the spermatodactyl with a non-sinuous duct (see above); and

(4) several other species have reticulate or lineate-reticulate epipleura that at least superficially resemble those of the *placentula* group of species. The ventral extent of the epipleura and its exact type of ornamentation should be scrutinized for each species. Members of the *placentula* group, *O.placentula*, *O.ussuriensis* and presumably *O.caucasicus* (note that *O.ussuriensis* and *O.caucasicus* were not illustrated dorsally) have a dorsal shield smooth or faintly reticulate, in contrast to conspicuously lineate-reticulate epipleura, which are relatively well extended ventrally (Table 2); this lineation-reticulation of the epipleura extends also anterodorsally to the region of setae j1–j2 and z1–z2. *Ololaelapsdililoensis* appears to have all diagnostic characters of the *placentula* group, but also has a clearly reticulate dorsal shield, at least in its posterior half. In *O.formidabilis*, the dorsal shield is only narrowly extending ventrally, but that region is conspicuously reticulated, in contrast (similarly to the *placentula* group) to the light, inconspicuous reticulation of the dorsal region of the shield.

*Ololaelapsburdwanensis*, *O.translineatus*, and *O.wangi* represent a cluster of very similar species. Finally, the last grouping in Table 2 (*O.bregetovae* and following species) may also represent a natural group, but given the intraspecific variation observed elsewhere (in *O.mooiensis*, see below; Table 2), it seems yet inappropriate to define a group based on the absence of fusion of shields (metapodal etc.) alone, especially given that all of these species need redescription.

The degree of fusion of the metapodal platelet with the various surrounding shields may vary significantly intraspecifically, as seen in *O.mooiensis* (incl. syn. *O.gamagarensis*; Nemati et al. 2018) where the metapodal platelet is exceptionally free from the hologastric shield in some individuals (Table 2). Some variation in the degree of fusion of the metapodal platelet with the hologastric shield also occurs in other species, such as *O.placentula* (Ryke 1962, Bregetova and Koroleva 1964, Evans and Till 1966); we have also observed, exceptionally, a specimen of that species with a metapodal platelet narrowly fused to the parapodal plate! Other examples are (1) *O.formidabilis*, having its metapodal platelet contiguous with, to indistinctly fused to, the hologastric shield (Fig. 3A, C–E), and (2) species in the *venetus* group, where the parapodal plate is clearly to ambiguously fused with the bridge (= metapodal) between the peritrematal and hologastric shields. Barilo (1991) also mentions that the ‘exopodal shields’ (= exopodal-parapodal) could be free or partly connected with the genitiventrianal shield in *O.translineatus*. Such intraspecific variation in shield fusion calls for caution when identifying species or sorting species into groups (as those presented in Table 2).

Intraspecific variation in shield fusions may occur in males too. For instance, some males that we identified as *O.placidus* have the peritrematal shield fused to the hologastric shield, just like the male of *O.venetus*, and others have the peritrematal shield free posteriorly, like that of the male of *O.sellnicki* (Bregetova 1977a).

At present, the chaetotaxy and the ornamentation of the dorsal shield are not clearly described for most *Ololaelaps* species (Table 2). At least some species (*O.formidabilis*; *O.placentula*, Evans and Till (1966); *O.mooiensis*, Marais and Loots (1972)) have a complete (or normal) dorsal chaetotaxy for a Laelapidae (sensu Evans and Till 1965). The illustrations of several other species indicate a slightly reduced dorsal chaetome. However, this should be verified, especially for setae apparently missing from marginal areas, in the r and S series, because these setae are difficult to discern in *Ololaelaps* species, which typically have slender setae and dark, heavily sclerotized dorsal shields. The presence of a single unpaired seta Jx is common in the genus; at least ten described species have it, four of which (*O.formidabilis*, *caucasicus*, *mooiensis*, *placidus*) have Jx present in some individuals, but absent in others. We suspect that this pliable character also varies in other species and that a Jx seta is expressed in some individuals only. The ornamentation of the dorsal shield is difficult to discern for species with light reticulation (e.g., *O.formidabilis*). Clearing the specimens thoroughly or slide-mounting some specimens dorsal side up should help; crushing selected specimens on the slide or dissecting their dorsal shield from the ventral idiosoma are other options.

Presently, differences in dimensions of the dorsal, sternal, and hologastric shields are only useful to separate species with marked differences, i.e., with elongate (e.g., *O.tasmanicus*) vs broad shields (e.g., *O.placentula*), because intraspecific variation is not sufficiently known. Ratios of length/width could be particularly useful, but they also vary intraspecifically, e.g., the sternal shield of *O.venetus* appears to have a length/width ratio of 0.8–1.0 (Bregetova and Koroleva 1964, Evans and Till 1966).

The position of seta st4 and poroid iv3 is difficult to use as a diagnostic character because it is not easy to determine whether they are on the shield margin, on the adjacent soft cuticle, or on the endopodal plate. This body region being the point of meeting of three shields (sternal, endopodal, hologastric) renders its study more difficult, obscuring the position of st4 and iv3, especially if they are inserted on soft cuticle, which can be folded above or underneath shields’ margins. Examining several specimens for each species can help, as well as making observations at different focal depths. We suspect that in most cases where st4 (and iv3) appears on the endopodal plate (e.g., *O.burdwanensis*, *O.sitalaensis*, *O.translineatus*), it is actually inserted on soft cuticle that overlaps the plate. Note that the position of st4 and iv3 are relatively stable within genera or even families of Gamasina, whether on soft cuticle, on metasternal platelets or (more rarely) on the sternal shield (e.g., Kazemi et al. 2008, Lindquist et al. 2009, Moraza and Linquist 2011).

The ornamentation of the hologastric shield shows species-specific patterns, such as inverted V or U-shaped ridges in *O.formidabilis* and undescribed species, as well as the shape of cells in the reticulation pattern (e.g., Barilo 1991). However, inter- and intraspecific variability needs to be ascertained, including for *O.placidus*, *O.venetus* and *O.sellnicki*. Bregetova and Koroleva (1964) and Bregetova (1977a) distinguished *O.sellnicki* from its close relative *O.venetus*, as well as *O.caucasicus* from *O.ussuriensis*, based on the hologastric shield having cells elongate transversally (*O.sellnicki*, *O.caucasicus*) vs regular cells or scales (*O.venetus*, *O.ussuriensis*). However, Evans and Till (1966) did not mention such distinction between *O.sellnicki* and *O.venetus*, perhaps because the distinction is not so straightforward. The cells of the reticulation also vary in shape, size, and conspicuousness (i.e., in the strength of the ridges) across the longitudinal (anterior to posterior) axis, and this ‘gradient’ may differ between species (Bregetova and Koroleva 1964). There is also interspecific differences in patterns of ridges on the sternal shields (Barilo 1991, Table 2; unpubl. data on undescribed species).

Our knowledge of the gnathosoma of *Ololaelaps* indicates limited variation between species. For instance, the internal malae have two pairs of projections in the females of all species where the hypostome has been described (*O.caucasicus*, *dililoensis*, *formidabilis*, *mooiensis*, *placentula*, *placidus*, *sellnicki*, *ussuriensis*, *venetus*, *wangi*) except for *O.sitalaensis* which lacks the lateral pair, based on the illustration in Bhattacharyya (1978). In contrast, the lateral pair of projections is missing in the males of all species where the hypostome have been described and the median projections are more fimbriate than those of females (*O.formidabilis*, Fig. 7; *O.sellnicki*, Bregetova and Koroleva 1964, Evans and Till 1966; *O.placidus*, unpubl. data). The number of rows of deutosternal denticles apparently varies at least intraspecifically (5–6 in *O.formidabilis*; 6–7 in *O.placidus*). On the other hand, there seems to be some interspecific variation in the number of denticles per rows, although often overlapping, with some species having six or fewer denticles per row (e.g., *O.formidabilis*, *ussuriensis*, *wangi*) and others having 5–10 denticles per row (*O.caucasicus*, *placentula*) (Bregetova and Koroleva 1964, Evans and Till 1966, Barilo 1991, Keum et al. 2017). Variation in cheliceral dentition is most notable for *O.interruptus* and *O.leptochelae* (see Table 2).

Idiosomal adenotaxy differs between laelapid species (Kazemi et al. 2014). Although the adenotaxy of only a few species of *Ololaelaps* has been studied, we have noticed variation in the position and shape of gland openings gd4 and gd9. This may prove to be useful in distinguishing species, especially as they often are easy to locate, being usually on or near the shield margin (on the ventrolateral extension) (e.g., figs 5, 13 in Bregetova and Koroleva (1964); Hassan 1989). Interestingly, the putatively related genus *Pseudoparasitus* has at least some members (Pseudoparasitussp. nearcentralis Berl.; unpubl. data) with gd4 and gd9 in similar positions, on the shield margin.

While the legs of *Ololaelaps* species mostly bear simple and slender setae, there is interspecific variation in the shape of setae. This should be investigated and exploited for species diagnostics (see examples in the genus description above).

## Supplementary Material

XML Treatment for Ololaelaps

XML Treatment for Ololaelaps formidabilis
